# Extensive intravesical benign hyperplasia induced by an extravesical migrated intrauterine device: A case report

**DOI:** 10.1097/MD.0000000000015671

**Published:** 2019-05-17

**Authors:** Peng Zhang, Ting Wang, Lu Yang

**Affiliations:** a Department of Urology; b Institute of Urology, West China Hospital, Sichuan University, Chengdu; c Department of Urology, The First People's Hospital of Yibin, Yibin, Sichuan, PR China.

**Keywords:** intrauterine device migration, tumor, urinary bladder

## Abstract

**Rationale:**

Intravesical migrated intrauterine devices (IUDs) have been reported to cause bladder perforation, stone formation, or malignant transition. However, such extensive intravesical benign hyperplasia caused by an extravesical migrated IUD is firstly reported.

**Patient concerns:**

A 38-year-old woman suffered from recurrent urinary urgency and dysuria and without macroscopic hematuria for about 1 month.

**Diagnoses:**

Urinary ultrasound and abdominal contrast-enhanced computed tomography (CT) revealed thickening of the bladder walls. Diagnostic transurethral resection and pathology initially misdiagnosed the intravesical lesions as non-invasive urothelial carcinoma. Further diagnostic and therapeutic transurethral resections and pathology confirmed the intravesical lesions to be extensive benign hyperplasia, which was extremely likely caused by the extravesical migrated IUD.

**Interventions:**

The intravesical lesions received therapeutic transurethral resections. Then the migrated IUD was removed by open surgery.

**Outcomes:**

After above treatments, the patient's lower urinary tract symptoms gradually disappeared. No recurrent lesion was found in the bladder through CT 3 months later.

**Lessons:**

Even an extravesical migrated IUD could silently cause extensive intravesical lesions. Whether symptomatic or not, any migrated IUD including extravesical and intravesical ones should be treated seriously, if possible, removed as soon as possible.

## 1 Introduction

Intrauterine devices (IUDs) are commonly used for reversible contraception worldwide.^[1]^ Although the migration of IUDs is infrequent, the condition may cause various complications including uterine perforation and/or damage to adjacent organs.^[2]^ The intravesical migrated IUDs reportedly led to bladder perforation,^[3]^ stone formation,^[4]^ or malignant transition.^[5]^ The present study firstly describes a case of a patient with extensive intravesical benign hyperplasia caused by chronic irritation of an extravesical migrated IUD.

## 2 Case report

**Figure 1:**
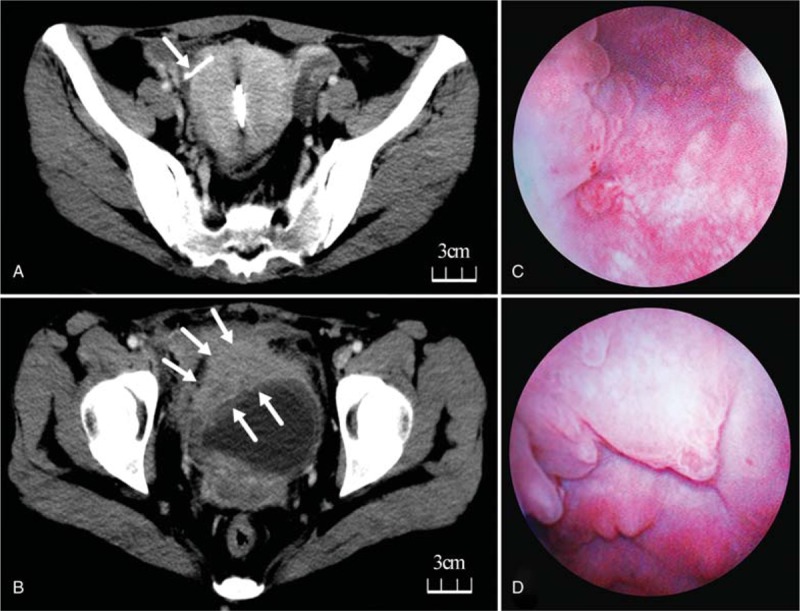
(a, contrast-enhanced CT): (1) Anterior bladder wall was extensively thickened (2.3 cm) and adjacent tissues were involved, (2) border of the posterior bladder wall could not be clearly distinguished from the uterus, (3) multiple lymph nodes were enlarged around the abdominal aorta, bilateral internal, and external iliac vessels (IUD was not mentioned by this CT report). (b, contrast-enhanced CT): A portion of one of the 2 implanted IUDs had migrated beyond the right uterus wall and was closely adjacent to the bladder. (c, d): Enormous intravesical benign hyperplasia were showed by cystoscopy.

A 38-year-old woman presented to a local hospital with a history of recurrent urinary urgency and dysuria and without macroscopic hematuria for 1 month. Urinalysis revealed leukocyturia (133/HP) and hematuria (25/HP). The patient was sexually active and was initially diagnosed with uncomplicated urinary infection. The patient received *norfloxacin* for 1 week. However, the symptoms remained unrelieved, and she was consulted for further examinations in the local hospital. As urinary ultrasound indicated thickening of the bladder anterior wall, further an abdominal contrast-enhanced computed tomography (CT) was carried out, through which more lesions were found, and malignant changes were highly suspicious (Fig. 1a). The cystoscopy from the primary hospital identified extensive basal mass in the bladder walls and the histological results of tissue biopsy revealed non-invasive urothelial carcinoma. Radical cystectomy was recommended by the provincial hospital owing to the extensive involvement of the bladder.

The patient considered the possibility of radical cystectomy to be devastating and presented at our hospital for consultation. A review of the patient's medical history revealed that the patient had undergone a Chinese IUD (Copper-bearing) placement 11 years ago after the birth of her first child. However, she became pregnant and underwent a painless induced abortion 3 months later, and the routine gynecological sonography revealed no IUD. After the following 2 accidental pregnancies, she underwent another IUD placement which worked well. Considering the above-mentioned history, our CT scanning revealed that a portion of one of the two implanted IUDs had migrated beyond the right uterus wall and was adjacent to the bladder (Fig. 1b). To verify the pathological diagnosis, diagnostic transurethral resection was performed (Fig. 1c, d), including the right, top, and trigone bladder wall, whereas histological examinations reported granuloma of the bladder right and top wall and glandular cystitis of the triangle wall. The result of the pathological analysis at the local hospital was sent to our pathology department for final confirmation, while result also revealed a benign granuloma.

As the results were controversial, a therapeutic (deeper and wider) transurethral bladder resection was performed in our hospital, and the pathology examination revealed the same benign conclusion. Finally, the uterus-IUD was removed at a gynecological clinic and the migrated IUD was removed by the cooperation of an urologist, gynecologist, and gastroenterologist. After the therapeutic transurethral bladder resection, the patient's lower urinary tract symptoms gradually disappeared. No recurrent lesion was noted in the bladder through computed tomography (CT) 3 months later (see Timeline, Supplemental Content, which illustrates the whole treatment process).

## 3 Discussion

IUDs are commonly used worldwide because of their reversible effects on contraception.^[1]^ Migration of IUD is infrequent but sometimes serious. Until now, more than 80 cases of IUDs migrated into the bladder have been described in PubMed. Which we can call the intravesical migrated IUDs, which caused bladder perforation,^[3]^ stone formation,^[4]^ or malignant transition.^[5]^ To our knowledge, this is the first report concentrating on an extravesical migrated IUD which induced extensive intravesical hyperplasia.

Migration of an IUD and uterine perforation occurs most frequently at the time of insertion,^[6]^ most likely be caused by nonstandard operations,^[7]^ so was in our case. A migrated IUD could cause chronic infections, which were believed as the etiological factors for malignant hyperplasia.^[5]^ Moreover, the copper-bearing IUD may continuously release Cu^2+^, which would promote chronic inflammatory response.^[8]^ As in our case, the intravesical lesions were likely to be resulted from chronic infection and irritation caused by the extravesical migrated copper-bearing IUD. Despite this hypothesis has to be verified in the future, we are happy to see that no intravesical recurrent lesion was found in 3 months after removing the migrated IUD.

Most of migrated IUDs cause lower urinary tract symptoms and could be easily diagnosed just by X-ray.^[9]^ Although clinical symptoms are not necessarily associated with the severity of the lesion, they guide the treatments. The management of a migrated IUD is controversial; however, its removal should be performed as soon as possible for symptomatic patients according to the World Health Organization and the International Medical Advisory Panel Meetings of the International Planned Parenthood Federation guidelines.^[4,9]^

The presence of severe intrapelvic adhesions may greatly increase the risks associated with surgery. While surgical operation would also increase intrapelvic adhesions. Therefore, some researchers recommend conservative treatment for asymptomatic patients.^[9]^ However, the above-presented case of our patient highlights certain interesting points.

With the advancement of auxiliary inspection technology and the increasing popularity of instruments, many doctors rely on auxiliary examinations and gradually ignore the medical history and physical examination. In the present study, the patient's medical history of the earlier lost IUD, which should have provided unparalleled clinical information for ultrasound doctors and radiologists, was ignored by grass-roots clinicians. Accurate pathological diagnosis is also crucial as a wrong diagnosis of non-invasive urothelial carcinoma would indicate a radical cystectomy, which is a terrible event for the 38-year-old patient.

But more importantly, our case taught us that an asymptomatic migrated IUD should not be ignored. An extravesical migrated IUD could also silently induce such extensive intravesical lesions. Whether the patient has symptoms or not, any migrated IUD including extravesical and intravesical one should be removed as soon as possible.

The presented case taught us that urologists should pay special attention to a patient's gynecological history as a detailed history is the basis of clinical practice. An extravesical migrated IUD could also silently cause extensive intravesical lesions. Whether causing symptoms or not, any migrated IUD including extravesical and intravesical ones should be treated seriously, if possible, removed as soon as possible.

## Author contributions

**Conceptualization:** Lu Yang.

**Data curation:** Peng Zhang, Ting Wang.

**Formal analysis:** Peng Zhang.

**Investigation:** Peng Zhang, Ting Wang.

**Resources:** Ting Wang.

**Software:** Peng Zhang.

**Writing – original draft:** Peng Zhang.

**Writing – review & editing:** Lu Yang.
